# Important differences between hypertensive middle-aged women and men in cardiovascular autonomic control—a critical appraisal

**DOI:** 10.1186/s13293-020-00355-y

**Published:** 2021-01-11

**Authors:** Stella V. Philbois, Tábata P. Facioli, Ada C. Gastaldi, Jhennyfer A. L. Rodrigues, Jens Tank, Thauane H. Fares, Karine P. Rodrigues, Hugo C. D. Souza

**Affiliations:** 1 Laboratory of Exercise Physiology and Cardiovascular Physiotherapy, Department of Health Sciences, Ribeirão Preto Medical School, University of São Paulo, Av. Bandeirantes, 3900, Vila Monte Alegre, Ribeirão Preto, SP 14049-900 Brazil; 2 Institute of Aerospace Medicine, German Aerospace Center, Cologne, Germany

**Keywords:** Sex, Hypertension, Cardiovascular autonomic control, Ovarian hormones

## Abstract

**Background:**

Normotensive premenopausal women show a vagal predominance of cardiac autonomic modulation, whereas age-matched men show a predominance of sympathetic modulation. However, some women develop systemic arterial hypertension (SAH) even with preserved ovarian function. Our hypothesis is that these women may have cardiovascular autonomic parameters similar to those of hypertensive men, even when subjected to pharmacological treatment. We aimed to investigate cardiovascular autonomic control and baroreflex sensitivity (BRS) in hypertensive premenopausal women and age-matched men.

**Methods:**

One hundred volunteers between 18 and 45 years of age were assigned to two groups (50 participants each): a hypertensive group including patients with a history of SAH for at least 6 months (25 men and 25 women), who were under treatment with monotherapy (losartan, 25–50 mg/kg); and a normotensive group (25 men and 25 women). Anthropometric, hemodynamic, metabolic, and autonomic cardiovascular assessments were performed focusing on BRS, autonomic modulation of heart rate variability (HRV), and blood pressure variability (BPV).

**Results:**

On HRV analysis, women showed higher values of high-frequency (HF) oscillations in absolute and normalized units, lower values ​of low-frequency (LF) in normalized units, and lower LF/HF ratio, as compared with men. When the normotensive and hypertensive groups were compared, hypertensive groups showed lower values ​of total variance and of LF and HF bands in absolute units. On BRS, hypertensive groups showed lower values than the normotensive group.

**Conclusion:**

Regardless of blood pressure control through pharmacological treatment, hypertensive patients continued to have reduced HRV compared to normotensive, and hypertensive men had more autonomic impairment than hypertensive premenopausal women.

## Introduction

Systemic arterial hypertension (SAH) is a multifactorial disease with a high prevalence in the adult population worldwide [1–3]. It is accompanied by impairments in cardiovascular homeostasis resulting from several factors, mainly endothelial dysfunction and changes in cardiac autonomic balance [4–6]. Autonomic changes are determined by an increased predominance of sympathetic autonomic components and/or a decrease in parasympathetic (vagal) components. If these are not controlled and/or reversed, the individual could be predisposed to a higher occurrence of acute myocardial infarction, cerebrovascular accident, and heart failure [1–3].

Among SAH patients, women have a lower prevalence of hypertension up until the climacteric period, compared with age-matched men. However, this occurrence is not observed after menopause [1–3]. The differences in prevalence between the sexes have been attributed, in part, to the action of ovarian hormones [7, 8], mainly estrogens. Estrogens play a key role in the release of dilating factors derived from the vascular endothelium as well as in cardiac autonomic control by promoting greater vagal autonomic participation and/or reduction of sympathetic influence on cardiac autonomic regulation [6, 7, 9, 10].

Despite the ovarian hormonal cardiovascular protection, some women precociously develop cardiovascular diseases, especially SAH. The causes of SAH before physiological ovarian failure are uncertain; however, it is possible that important changes in cardiovascular autonomic control might play a role. It is well known that cardiac autonomic balance regulation is different between the sexes; while women are characterized by a predominant vagal autonomic component, there is sympathetic predominance in men, especially with relation to heart rate variability (HRV) modulation [10–12]. In addition, it is possible that SAH and its complications may cancel out the cardiovascular protective effect of ovarian hormones due to there being no sex differences in pathophysiological cardiovascular autonomic modulation such as those reported in acute myocardial infarction and heart failure [13–15], contrary to what is observed in normotensive individuals [10, 11]. However, most studies have been carried out on women post menopause, a period known to be characterized by substantial reduction in HRV and baroreflex sensitivity (BRS) [7]. Therefore, we investigated cardiovascular autonomic control in hypertensive men and in premenopausal women, focusing on BRS and the balance of cardiovascular autonomic modulation.

## Methods

One hundred volunteers between 18 and 45 years of age were assigned to two groups (fifty participants each): hypertensive group—patients with a 0.5–3-year history of SAH—(25 men and 25 premenopausal women) and normotensive group (25 men and 25 women). All volunteers were screened at the Laboratory of Exercise Physiology and Cardiovascular Physiotherapy (LAFFIC) of the Ribeirão Preto Medical School (FMRP-USP). The hypertensive volunteers had a history of essential arterial hypertension stages I and II, with low to moderate cardiovascular risk [2]. All hypertensive volunteers had been treated with only monotherapy using AT_1_ receptor blocker (losartan, 25–50 mg/day) for at least 6 months. The inclusion criteria comprised the absence of the following: smoking, cognitive disturbances, pregnancy, use of hormonal contraceptives, musculoskeletal disorders, cardiovascular diseases (except for SAH), and any other disease that compromised the assessments. This study was approved by the Ethics Committee of the Ribeirão Preto Medical School’s Hospital (HCFMRP/USP) under protocol number 2.791.486/2018. The volunteers were informed of the ethical and legal aspects of the study and signed the free and informed consent form prior to the start of the study.

### Protocols

Data were collected in the morning during two laboratory visits, between 07:00 and 10:00 am, with a 48-h interval between visits. All women had a regular menstrual cycle, and all the data were collected in the follicular phase of their cycle. The first assessment included anthropometric measurements and blood collection at the Laboratory of the Clinical Research Support Centre of the Ribeirão Preto Medical School’s Hospital (HCFMRP/USP).

The second visit took place at the Laboratory of Exercise Physiology and Cardiovascular Physiotherapy of the Ribeirão Preto Medical School. The volunteers had their heart rate and blood pressure recorded in the supine position, followed by cardiorespiratory function test on a motorized treadmill. Each visit lasted approximately 2 h.

All volunteers were asked to avoid drinking alcoholic beverages and instructed to maintain their usual diet for 48 h prior to the assessments. They were also instructed to maintain their regular medication intake (losartan, 25–50 mg) and advised to sleep for at least 7−8 h on the night before the assessments.

### Laboratory exams

Blood samples (3.5 mL, BD Vaccutainer® EDTA; Becton, Dickin, and Company, Franklin Lakes, NJ, USA) was used to analyze levels of fasting glycemia (hexokinase-UV), triglycerides (desidrogenase), total cholesterol and its fractions (esterase-oxidase), estrogen (chemiluminescence), and testosterone (radioimmunoassay). All volunteers were asked to fast for 12 h prior to the assessments.

### Anthropometry and body composition

Evaluations followed the recommendations of the International Society for the Advancement of Kinanthropometry. The weight and height values were obtained using the Welmy analog scale with an altimeter (Welmy, Santa Bárbara d’Oeste, São Paulo, Brazil). The body mass index (BMI) of the volunteers was calculated as weight/height^2^, with weight in kilograms and height in meters.

To evaluate the body fat percentage, an experienced evaluator performed the measurements of skin folds. The adipometer used was the Sanny®, with a 0.1-mm scale, mandibular pressure of 9.8 g/mm^2^, and contact area (surface) of 97 mm^2^, according to the manufacturer’s specifications. The skin folds were evaluated at the following points: triceps, subscapular area, axilla (midaxillary), supra iliac area, abdomen, thigh, and chest. From the data obtained, the following parameters were calculated: sum of the seven skin folds (ΣSF) (mm) and the body fat percentage (% BF), estimated according to the age group [16].

### Cardiorespiratory function test

An incremental treadmill exercise test (Super ATL Millenium®, Inbramed/Inbrasport, Porto Alegre, RS, Brazil) was performed with a submaximal test established with the HR corresponding to the sum of the baseline HR plus 85% of the reserve HR (maximum HR − basal HR), following the previously described Balke protocol [17]. Electrical activity was monitored by an electrocardiogram (ECG) with nine leads (CM5, DI, DII, V1-V6). Oxygen and dioxide carbon uptake (VO_2_ and VCO_2_, respectively) were obtained using a metabolic analyzer (Ultima™ CardiO2, Medical Graphics Corp., St. Paul, Minneapolis, USA).

### Hemodynamic assessment

Systolic blood pressure (SBP), diastolic blood pressure (DBP), and mean blood pressure (MBP) were obtained using the Finometer equipment (Finometer Pro, Finapres Medical System, Amsterdam, Netherlands). Heart rate (HR) data were obtained using an electrocardiographic digital recorder (ML866 PowerLab, ADInstruments, Bella Vista, Australia).

### Heart rate variability and blood pressure variability analysis

Heart rate variability (HRV) data were obtained using the RR intervals (iRR) from the electrocardiographic record (ECG) through the modified CM5 shunt, at a sampling frequency of 2000 Hz. Blood pressure variability (BPV) values were obtained from the beat-to-beat SBP data using digital plethysmography recording equipment (Finometer Pro, Finapres Medical System, Amsterdam, Netherlands), with a cuff positioned on the middle finger of the right upper limb. The data interface to the microcomputer was performed using the PowerLab4/35 device (ADInstruments, Australia). The data were recorded and stored (Software LabChart 8.0, ADInstruments, Australia) for further analysis. Volunteers were instructed to remain in the supine position for approximately 10 min to stabilize cardiovascular parameters. After this period, the ECG and arterial pulse pressure were recorded simultaneously for another 10 min. The temperature (22 °C) and ambient lighting were controlled, and the sessions were performed in a noise-free environment.

For the standardization of biological signal acquisition and recording, the BP plethysmography recording equipment was calibrated before each test using physiological calibration and return-to-flow (RTF) in addition to the photoplethysmography height sensor. This procedure allowed for adjustment of the peripheral pressure values (cuff on the middle finger) as compared to the brachial artery pressure values (cuff positioned on the upper region of the ipsilateral arm). Uninterrupted biological signals were recorded after this calibration phase. BPV and HRV analyses were performed using custom computer software (CardioSeries v2.4, http://sites.google.com/site/cardioseries) developed by Dias, DPM of the University of São Paulo, Brazil [18]. The values of RRi and SBP intervals were redesigned in a 3-Hz cubic spline interpolation to normalize the time interval between the beats. The series of interpolated RRi and SBP were divided into half-overlapping sets of 256 data points, overlapping 50% (Welch Protocol). The stationary segment was visually inspected, and those with artifacts or transients were excluded. Each RRi and SBP stationary segment was subjected to spectral analysis by fast Fourier transform (FFT), after the Hanning window. RRi time series were integrated in bands of low frequency (LF; 0.04–0.15 Hz) and high frequency (HF; 0.15–0.5 Hz), and the results were expressed in absolute (ms^2^) and normalized units (nu), whereas the SBP time series were integrated only in the low-frequency band (LF; 0.04–0.15 Hz), and the results were expressed in absolute values (mmHg^2^).

The normalized values for HRV were obtained by calculating the percentage of LF and HF power in relation to the total spectrum power minus the very low-frequency band (VLF; < 0.04 Hz). In addition, the normalization procedure was performed to minimize the total power variations in the absolute values of LF and HF [19]. To assess the sympathovagal balance, the LF/HF ratio of RRi variability was also calculated [20].

### Spontaneous baroreflex sensitivity analysis

BRS was assessed in the time domain using the sequence technique [21]. The computer software CardioSeries v2.4 scanned the beat-to-beat time series of pulse interval (PI) and SBP values, searching for sequences of at least 3 consecutive beats, in which progressive increases in SBP were followed by progressive increases in PI (up sequence) and progressive decreases in SBP were followed by progressive decreases in PI (down sequence), with a correlation coefficient (*r*) between PI and SBP (values higher than 0.8). Spontaneous BRS was determined by the mean slope of the linear regression line between the SBP and PI values of each sequence. The number of baroreflex sequences found (per 1000 beats) and the mean individual slope of the significant SAP/PI relationship, obtained by averaging all slopes computed within the test period, were calculated and used as a measure of spontaneous BRS.

The sequence method also presents the baroreflex effectiveness index (BEI). It is the ratio of the number of sequences and the total number of SBP ramps. The BEI shows how many SBP changes are effectively translated into a change in PI, independent of its magnitude [22].

### Statistical analysis

Electronic spreadsheets were prepared, and the information was analyzed using the electronic program Sigma-Stat®, version 11. Variables were analyzed using parametric and non-parametric tests, when required. The effects of sex and hypertension were analyzed by two-way ANOVA and, when appropriate, a post hoc comparison was performed using the Student-Newman-Keuls method. Student’s *t* test was used for comparisons between groups. The *P* value was set at ≤ 0.05.

## Results

Table 1 shows the characteristics and hemodynamic parameters of the groups. Women in both groups had lower body weight and height than men. However, BMI and body fat percentage were similar between men and women. With respect to hemodynamic and cardiorespiratory fitness parameters, men showed higher values ​of VO_2peak_, systolic, diastolic, and mean blood pressure when compared to women. However, when hypertensive and normotensive groups were compared, the patients in the hypertensive groups showed higher systolic and mean arterial pressures. Women had higher estrogen, lower testosterone, and lower T/E ratios than men. Women also had lower values ​of fasting blood glucose, triglycerides, LDL and total cholesterol, and higher values of HDL when compared to men, as shown in Table 2. On the other hand, when hypertensive and normotensive groups were compared, fasting blood glucose values ​were higher in the hypertensive volunteers.

**Table 1:** Hemodynamic and characteristics parameters of all groups

	Women		Men		Sex factor		Hypertension factor		Interaction	
	Normotensive	Hypertensive	Normotensive	Hypertensive	*F*_(d.f)_	*P*	*F*_(d.f)_	*P*	*F*_(d.f)_	*P*
**Characteristics**										
Age, years	40 ± 4	42 ± 3	41 ± 3	42 ± 4	*F*_(1,99)_:0.47	0.493	*F*_(1,99)_:1.9	0.165	*F*_(1,99)_:0.14	0.703
Weight, kg	71 ± 6	73 ± 6	84 ± 9	86 ± 7	*F*_(1,99)_:73.5	< 0.001	*F*_(1,99)_:2.9	0.09	*F*_(1,99)_:0.06	0.798
Height, m	1.65 ± 0.06	1.65 ± 0.05	1.77 ± 0.06	1.75 ± 0.08	*F*_(1,99)_:64.9	< 0.001	*F*_(1,99)_:2.1	0.150	*F*_(1,99)_:0.68	0.411
BMI, kg/m^2^	26 ± 3	27 ± 3	27 ± 3	28 ± 3	*F*_(1,99)_:2.6	0.108	*F*_(1,99)_:0.7	0.389	*F*_(1,99)_:0.03	0.854
Body fat percentage	23 ± 3	25 ± 3	23 ± 3	24 ± 4	*F*_(1,99)_:0.09	0.766	*F*_(1,99)_:2.9	0.093	*F*_(1,99)_:0.91	0.343
**Hemodynamic parameters**										
VO_2peak_, ml/kg/min	29 ± 3	28 ± 4	32 ± 5	30 ± 5	*F*_(1,99)_:6.9	0.010	*F*_(1,99)_:2	0.105	*F*_(1,99)_:0.07	0.791
HR, bpm	78 ± 11	77 ± 9	75 ± 11	75 ± 7	*F*_(1,99)_:0.02	0.904	*F*_(1,99)_:0.13	0.718	*F*_(1,99)_:1.66	0.200
SBP, mmHg	108 ± 11	118 ± 18	121 ± 12	124 ± 15	*F*_(1,99)_:10.4	0.002	*F*_(1,99)_:5.4	0.022	*F*_(1,99)_:1.97	0.164
DBP, mmHg	72 ± 10	75 ± 8	79 ± 9	82 ± 11	*F*_(1,99)_:11.7	< 0.001	*F*_(1,99)_:2.8	0.096	*F*_(1,99)_:0.02	0.891
MBP, mmHg	84 ± 10	90 ± 11	93 ± 8	99 ± 12	*F*_(1,99)_:19.1	< 0.001	*F*_(1,99)_:8.1	0.006	*F*_(1,99)_:0.01	0.913

Data presented as mean ± SD

*BMI* body mass index, *W/H* waist/hip ratio, *VO*_*2peak*_ oxygen consumption at peak of exercise, *HR* heart rate, *SBP* systolic blood pressure, *DBP* diastolic blood pressure, *F* factor, *d.f.* degrees of freedom

**Table 2:** Laboratory tests of all groups

	Women		Men		Sex factor		Hypertension factor		Interaction	
	Normotensive	Hypertensive	Normotensive	Hypertensive	*F*_(d.f)_	*P*	*F*_(d.f)_	*P*	*F*_(d.f)_	*P*
Estrogen, pg/mL	85.4 ± 19.7	80.4 ± 24.4	15.7 ± 4.5	15.4 ± 6.3	*F*_(1,99)_:433.4	< 0.001	*F*_(1,99)_:0.7	0.416	*F*_(1,99)_:0.53	0.467
Testosterone, ng/mL	0.94 ± 0.2	0.87 ± 0.3	7.1 ± 1.7	6.4 ± 1.5	*F*_(1,99)_:630.5	< 0.001	*F*_(1,99)_:2.3	0.134	*F*_(1,99)_:1.41	0.238
T/E, pg/mL	11.8 ± 4.7	12.1 ± 6.5	490 ± 179.7	479 ± 194.7	*F*_(1,99)_:317.4	< 0.001	*F*_(1,99)_:0.04	0.834	*F*_(1,99)_:0.05	0.824
Glucose, mg/dL	87.4 ± 8.5	93.2 ± 14	90.3 ± 6.1	106.7 ± 13.5	*F*_(1,99)_:13.7	< 0.001	*F*_(1,99)_:25.2	< 0.001	*F*_(1,99)_:5.64	0.020
Triglycerides, mg/dL	101.9 ± 10.9	92.6 ± 37.6	144.3 ± 31.9	165.7 ± 95.4	*F*_(1,99)_:28.6	< 0.001	*F*_(1,99)_:0.3	0.578	*F*_(1,99)_:2.02	0.158
Total Cholesterol, mg/dL	193.9 ± 16.4	189.8 ± 16.7	195.4 ± 9.7	208.6 ± 42.6	*F*_(1,99)_:4.2	0.044	*F*_(1,99)_:0.8	0.364	*F*_(1,99)_:3.1	0.083
HDL, mg/dL	49.1 ± 6.7	53.1 ± 8.5	47.1 ± 8.4	39.2 ± 8.1	*F*_(1,99)_:20.0	< 0.001	*F*_(1,99)_:1.6	0.213	*F*_(1,99)_:13.5	< 0.001
LDL, mg/dL	114.1 ± 11.5	116.2 ± 17.5	125.3 ± 11.2	134.1 ± 33.7	*F*_(1,99)_:12.3	< 0.001	*F*_(1,99)_:1.8	0.183	*F*_(1,99)_:0.63	0.428

Data presented as mean ± SD

*pg/mL* picogram per milliliter, *ng/mL* nanogram per milliliter, *mg/dL* milligrams deciliters, *T/E* ratio between testosterone and estrogen, *HDL* high-density lipoprotein, *LDL* low-density lipoprotein, *F* factor, *d.f.* degrees of freedom

**Fig. 1:**
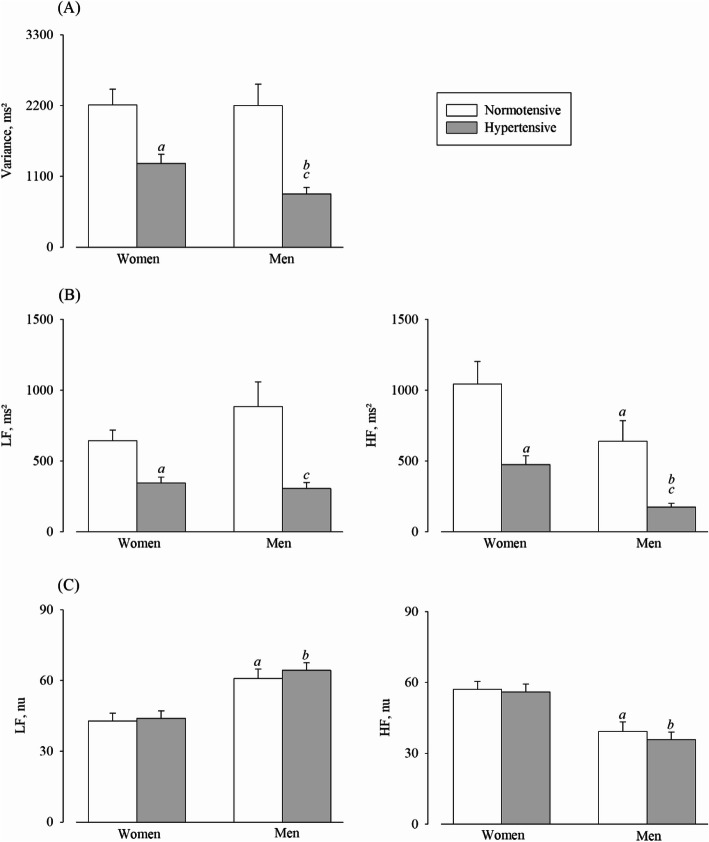
Heart rate variability in supine position. **a** Total variance of the spectral density. **b** Spectral power density of heart rate in absolute units (ms^2^) in low (LF) and high frequency (HF). **c** Spectral power density of heart rate in the LF and HF bands in normalized units (nu). Values are presented as the mean ±SEM. ^a^*P* ≤ 0.05 versus normotensive women group; ^b^*P* ≤ 0.05 versus hypertensive women group; ^c^*P* ≤ 0.05 versus normotensive men group

As shown in Table 3 and Fig. 1, on comparing women and men, the HRV analysis obtained in the supine position showed that women had higher values ​of HF oscillations in absolute and normalized units as well as lower values ​of LF in normalized units and LF/HF ratio. When the normotensive and hypertensive groups were compared, hypertensive women and men showed lower values ​of total variance and of LF and HF bands in absolute units. There were no differences related to sex or hypertension for BPV; however, hypertensive men and women showed lower BRS values (baroreflex gain, ms/mmHg) when compared to normotensive volunteers (Table 3). Additionally, we also observed that hypertensive men had lower BRS values when ​compared to hypertensive women.

**Table 3:** Volunteer’s cardiovascular autonomic parameters evaluated at rest and supine positions

	Women		Men		Sex factor		Hypertension factor		Interaction	
	Normotensive	Hypertensive	Normotensive	Hypertensive	*F*_(d.f)_	*P*	*F*_(d.f)_	*P*	*F*_(d.f)_	*P*
**Heart rate variability**										
RRi	784 ± 23	793 ± 13	810 ± 25	953 ± 74	*F*_(1,99)_:1.1	0.300	*F*_(1,99)_:0.04	0.840	*F*_(1,99)_:0.06	0.813
Variance, ms^2^	2209 ± 1219	1303 ± 697	2199 ± 1657	829 ± 493	*F*_(1,99)_:1.2	0.279	*F*_(1,99)_:26.1	< 0.001	*F*_(1,99)_:1.08	0.300
LF, ms^2^	643 ± 371	344 ± 206	885 ± 866	305 ± 215	*F*_(1,99)_:1.05	0.308	*F*_(1,99)_:19.7	< 0.001	*F*_(1,99)_:2.01	0.159
HF, ms^2^	1044 ± 801	474 ± 320	635 ± 726	173 ± 148	*F*_(1,99)_:9.6	0.003	*F*_(1,99)_:20.7	< 0.001	*F*_(1,99)_:0.21	0.649
LF, nu	43 ± 17	44 ± 16	61 ± 21	64 ± 16	*F*_(1,99)_:30.2	< 0.001	*F*_(1,99)_:0.4	0.512	*F*_(1,99)_:0.12	0.736
HF, nu	57 ± 17	56 ± 16	39 ± 21	36 ± 16	*F*_(1,99)_:30.2	< 0.001	*F*_(1,99)_:0.4	0.512	*F*_(1,99)_:0.12	0.736
LF/HF ratio	0.91 ± 0.6	0.99 ± 0.9	2.35 ± 2	2.52 ± 1.9	*F*_(1,99)_:24.1	< 0.001	*F*_(1,99)_:0.2	0.669	*F*_(1,99)_:0.02	0.900
**Blood pressure variability**										
Variance, mmHg^2^	24 ± 18	25 ± 9.52	19 ± 12	27 ± 18.7	*F*_(1,99)_:0.3	0.581	*F*_(1,99)_:2.2	0.139	*F*_(1,99)_:1,28	0.260
LF, mmHg^2^	7 ± 6.2	6 ± 2.7	6 ± 4.8	8 ± 5.8	*F*_(1,99)_:0.45	0.496	*F*_(1,99)_:0.09	0.758	*F*_(1,99)_:2.1	0.150
**Baroreflex sensitivity**										
Baroreflex sequences	87 ± 20	98 ± 14	85 ± 16	76 ± 48	*F*_(1,99)_:4.5	0.036	*F*_(1,99)_:0.02	0.895	*F*_(1,99)_:3.26	0.074
BEI	0.73 ± 0.16	0.61 ± 0.13	0.64 ± 0.14	0.47 ± 0.19	*F*_(1,99)_:14.1	< 0.001	*F*_(1,99)_:21.3	< 0.001	*F*_(1,99)_:0.82	0.366
Gain, ms/mmHg	14.5 ± 5.7	8.7 ± 4.1	13.1 ± 9.7	6.6 ± 2.7	*F*_(1,99)_:2.1	0.153	*F*_(1,99)_:24.7	< 0.001	*F*_(1,99)_:0.10	0.749
Up, ms/mmHg	13.8 ± 5.9	8.4 ± 3.9	12.7 ± 10.1	6.5 ± 3	*F*_(1,99)_:1.4	0.245	*F*_(1,99)_:21.1	< 0.001	*F*_(1,99)_:0.10	0.752
Down, ms/mmHg	14.8 ± 5.6	9.1 ± 4.5	13.4 ± 0.6	6.7 ± 2.5	*F*_(1,99)_:2.5	0.120	*F*_(1,99)_:25.6	< 0.001	*F*_(1,99)_:0.18	0.673

Data presented as mean ± SD

*RRi* R-R intervals, *ms* milliseconds, *LF* low-frequency band, *HF* high-frequency band, *nu* normalized units, *mmHg* mercury millimeters, *BEI* baroreflex effectiveness index, *ms/mmHg* milliseconds/millimeter of mercury, *up* baroreflex sequence with progressive increases in blood pressure followed by progressive increases in pulse interval, *down* baroreflex sequence with progressive decreases in blood pressure followed by progressive decreases in pulse interval, *F* factor, *d.f.* degrees of freedom

## Discussion

Cardiovascular autonomic control has been widely studied in different pathophysiological conditions, and these studies have guided diagnoses and treatments, and been used as predictors of cardiovascular morbidity and mortality [19, 23]. For SAH, an increase in sympathetic participation and/or reduction of vagal participation in cardiac autonomic balance is frequently observed in the several parameters evaluated [4, 5]. In most cases, these changes result in a reduction in BRS [4, 24]. In turn, when indices that quantify cardiac autonomic modulation are used, it is common to observe a decrease in HRV, and together with a reduction in BRS, they indicate an important loss of autonomic mechanisms in cardiovascular protection.

On the other hand, although normotensive adult men and women share the same autonomic mechanisms of cardiovascular autonomic regulation, they show a significant difference in cardiac autonomic modulation regulation. In this case, women have a vagal predominance of cardiac autonomic modulation, while men have a predominance of sympathetic modulation [10, 11]. However, little is known about these differences in pathophysiological conditions, such as cardiovascular diseases. Thus, it is common for studies to include men and women, without considering these sex-related particularities of cardiac autonomic control. In this case, our results showed that women and men with SAH have differences in cardiac autonomic regulation; specifically, a predominance of vagal autonomic modulation was seen in women and sympathetic autonomic modulation in men. However, the absolute HRV values found in the present study were much lower than those in the normotensive groups, mainly regarding the HF oscillations in the men. In this case, the results corroborate the statement that SAH leads to HRV reduction, an important autonomic change. Interestingly, the reduction in HRV did not affect the balance of autonomic modulation; therefore, the predominance was similar to that observed in normotensive women and men. The reason for maintaining the vagal predominance in the cardiac autonomic balance in hypertensive women is uncertain; however, sexual characteristics, mainly sexual female hormones, might play a role in this. Estrogens might be the main hormones at play, since experimental and clinical studies have shown that they play an important role in autonomic regulation, increasing vagal autonomic modulation, and reducing cardiac sympathetic autonomic modulation [7]. Furthermore, other studies indicate that estrogens might influence vascular tone regulation and, consequently, arterial blood pressure by different mechanisms such as the release of vasodilating factors from the endothelium, antioxidant and anti-inflammatory factors [6, 25, 26], downregulation of angiotensin-converting enzyme [27], and reduced activity of angiotensin II (AT1) receptors [28], which together have a positive influence on cardiac contractility [9]. Corroborating this information, our results showed that women had lower BP values, even if they underwent identical pharmacological treatment (losartan). On the other hand, aging is associated with a higher prevalence of SAH, including in women. In this case, the deprivation of ovarian hormones stands out as the main risk factor because after menopause, the prevalence of SAH between the sexes is similar [1–3]. With regard to cardiovascular autonomic control, the literature shows that after menopause, normotensive women have a significant reduction in HRV. This reduction is largely attributed to the deprivation of ovarian hormones [7, 29].

In turn, studies in the literature have shown that male hormones also influence cardiovascular autonomic control [30]. In men, an excess of androgens seems to cause vagal cardiac dysfunction, in addition to disturbances in ventricular regulation [31]. Another important observation on the differences in cardiac autonomic modulation between the sexes can be exemplified in women with polycystic ovary syndrome. In this group of women, there is a significant increase in the main male hormone testosterone, which is positively correlated with an increased influence on the sympathetic autonomic component [32]. Thus, testosterone levels in men and estrogens in women are variables that could explain the sex differences found in the autonomic modulation of HRV, including in patients with SAH.

Other aspects that may influence the predominance of sympathetic autonomic modulation in men involve body composition and the investigated blood parameters. We know that men have a higher volume of skeletal muscle mass, which may result in greater participation of the sympathetic autonomic component in cardiovascular regulation [33]. The literature also shows that men have a greater number of sympathetic ganglion neurons, than women [34]. In our study, despite the similar anthropometric results, men tended to have higher BMI and lower body fat percentage, suggesting that this group had a higher percentage of lean mass. On the other hand, blood parameters were quite different. Women had higher values of HDL and lower values of triglycerides and fasting glucose. In fact, these differences could justify the reduction of autonomic modulation in hypertensive men, but not in hypertensive women.

Two findings in our study are intriguing and point to the importance of the different hormonal characteristics of the sexes. The first is the significant reduction in the values of HRV vagal autonomic modulation in hypertensive men, characterized by HF component oscillations (men, 173 ± 30 ms^2^; women, 474 ± 64 ms^2^). While hypertensive women showed a reduction by half (normotensive, 1044 ± 160 ms^2^; hypertensive, 474 ± 64 ms^2^), men showed a reduction by almost 1/5 (normotensive, 635 ± 146 ms^2^; hypertensive 173 ± 30 ms^2^). In this case, it is possible that this reduction is related to a lower BRS. It should be noted that in the normotensive group (men and women), there were no differences in BRS. All this indicates that SAH appears to induce greater damage in men, as evidenced by a significant reduction of vagal participation in cardiovascular autonomic regulation. The second finding is that during treatment with losartan, despite blood pressure (BP) normalization, the cardiac autonomic modulation parameters were well below the desirable level, as noted above. This is worrisome, since HRV indices are often used as morbidity and mortality predictors [19]. However, as our findings demonstrate, men are less protected, mainly because they exhibit an excessive drop in HF oscillations that correspond to vagal autonomic modulation. Finally, the significant reduction in the HF component of HRV may also be due, at least in part, to the metabolic alteration found in hypertensive men.

## Perspectives and significance

Hypertensive women and men, even when treated with losartan, show reductions in HRV, when compared to normotensive volunteers. Despite systemic arterial hypertension, premenopausal women and age-matched men have a different predominance of autonomic components over cardiac modulatory balance. Premenopausal women are more dependent on vagal modulation, whereas men have a greater prevalence of the sympathetic autonomic component. These findings suggest that men have higher cardiovascular risks and that the characteristics of sex hormones are the possible causes of the differences observed between men and women. However, further studies are needed to identify the precise mechanisms responsible for these findings.

## Data Availability

The datasets during and/or analyzed during the current study are available from the corresponding author on reasonable request.
